# More data on erythema annulare centrifugum induced by COVID‐19 vaccination

**DOI:** 10.1111/dth.15629

**Published:** 2022-06-21

**Authors:** Alexander Kreuter, Jimmy Jos Puthussery, Julia Hyun, Valentina Laura Müller

**Affiliations:** ^1^ Department of Dermatology, Venereology, and Allergology, Helios St. Elisabeth Hospital Oberhausen University Witten‐Herdecke Witten Germany; ^2^ Department of Dermatology, Venereology, and Allergology Helios St. Johannes Hospital Duisburg Germany

Dear Editor,

Erythema annulare centrifugum (EAC) is a rare skin disease appearing as recurrent erythematous annular eruptions. Recently, a case of EAC induced by the adenovirus‐vectored vaccine AZD1222 (ChAdOx1 nCov‐19) was reported.^1^ So far, more than 11 billion vaccine doses against SARS‐CoV‐2 have been administered worldwide. The number of vaccine‐induced cutaneous side‐effects reported in the literature is continuously increasing, and most SARS‐CoV‐2‐associated comprise local side reactions, delayed large local side reactions, urticaria, morbilliform exanthema, erythromelalgia, and reactivation of viral infections^2^, ^3^ We would like to add a second patient with recurring episodes of EAC following mRNA‐based vaccination with BNT162b2 as well as administration of the first and second booster dose. Experimental subcutaneous injection of BNT162b2 in this patient resulted in a sudden deterioration of EAC.

A 55‐year‐old woman presented with multiple intensely itching, annular erythematous plaques located at both legs. She reported that first skin lesions occurred 10 days after the second dose of COVID‐19 vaccination with BNT162b2. Treatment with levocetericine and topical prednicarbate cream completely resolved all skin lesions and pruritus within 14 days. Following booster vaccination 3 months later, a new but more extensive rash occurred at both lower legs. Tapering systemic corticosteroids, beginning with 150 mg prednisone and topical predicarbate cream were initiated, and again cleared her skin lesions within 3 weeks. The patient presented for the first time in our department with a further onset of annular plaques after the second booster vaccination in April 2022 (Figure 1A). Histopathological analysis of a representative lesion revealed discrete spongiosis, parakeratosis and a tight lymphohistiocytic infiltrate surrounding superficial vessels. Moreover, some isolated fungal hyphae were detected, most likely indicating secondary fungal colonisation. Based on these findings, a diagnosis of EAC was made. Laboratory investigations found a normal blood cell count and serum chemistry, and normal titres for antinuclear antibodies and extractable nuclear antigens. Further work‐up including chest X‐ray, abdominal ultrasound, and heart echography was unremarkable.

**FIGURE 1:**
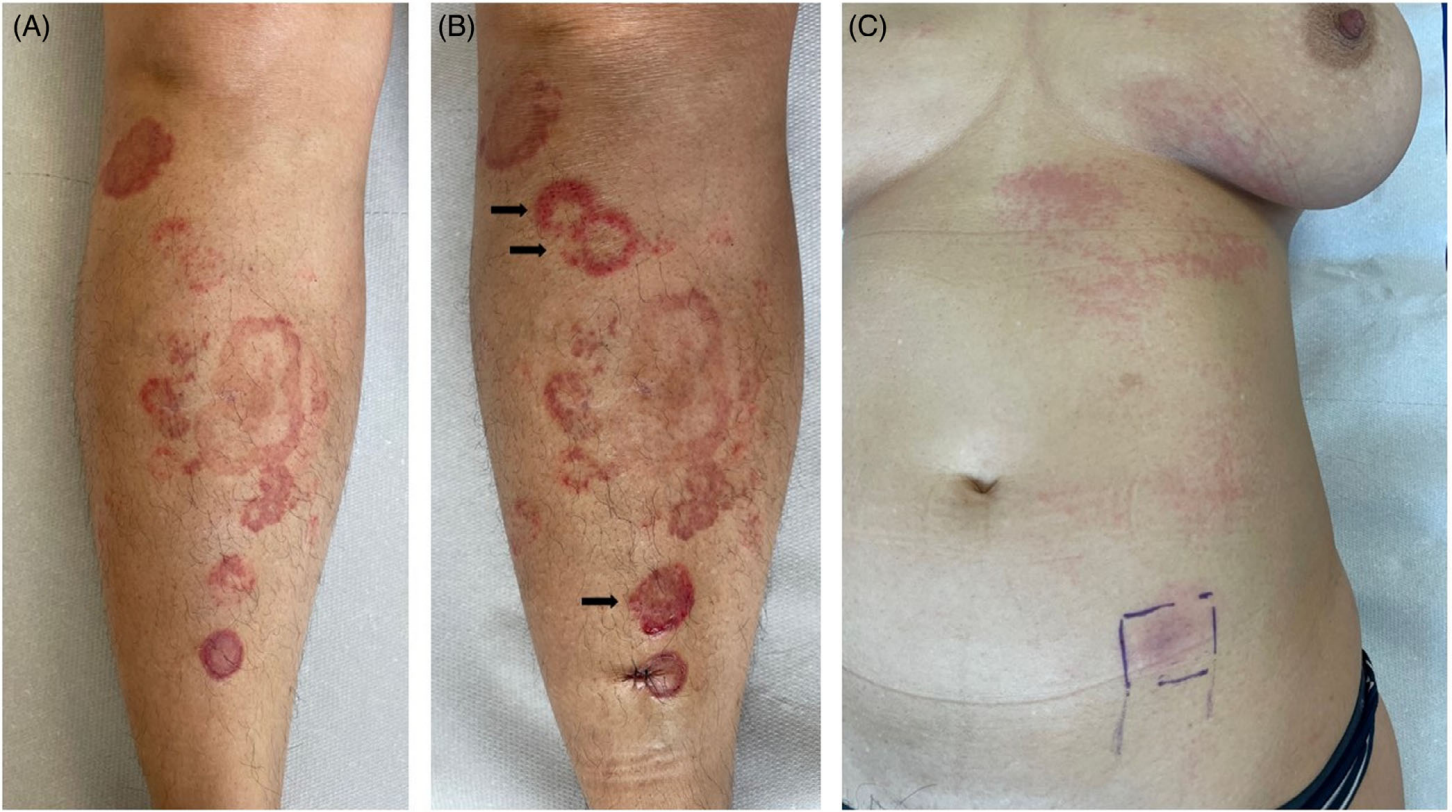
(A) Clinical findings at first presentation in our department. Ten days after the second booster vaccination with BNT162b2, multiple annular erythematous patches and plaques with a central pale zone occurred at the lower legs. In some lesions, scaling is present inside the advancing edge (called trailing scale). (B) Deterioration of erythema annulare centrifugum (see black arrows) 48 h after experimental subcutaneous injection of 0.1 ml BNT162b2. (C) Maculopapular exanthema of the trunk occurring approximately 3 h after experimental subcutaneous injection of BNT162b2

In order to evaluate a cause‐effect relationship of EAC with the COVID‐19 vaccination, we injected 1 ml of BNT162b2 (30% of the standard‐dose) subcutaneously at the lower abdomen. This induced a pruritic maculopapular rash on the trunk occurring approximately 3 h after injection, and also led to a deterioration of EAC lesions at the lower legs 2 days later (Figure 1B,C).

EAC, a rare type of gyrate erythema, is clinically characterized by papules and plaques that expand centrifugally, resulting in characteristic annular lesions.^4^ EAC is considered to be a hypersensitivity reaction to a wide range of potential triggers, including infections, malignancies, foods, drugs, and vaccines.^5^ Interestingly, both immediate (type I) and delayed (type IV) hypersensitivity reactions have been reported in association with COVID‐19 vaccination.^2^ Other possible pathomechanisms of SARS‐CoV‐2 induced cutaneous lesions include mechanisms of molecular mimicry caused by genetic similarities between SARS‐CoV‐2 spike protein components to cross‐reactive human antigens leading to autoreactive lymphocytes or cross‐reactive human antigens, functional angiopathies, or reactivation of viral infections.^6^

Although simultaneous occurrence does not prove a causal connection, the recurrent onset of EAC after each vaccination (including both boosters) as well as immediate maculopapular rash following low‐dose subcutaneous BNT162b2 injection are highly suggestive for a vaccine‐induced unspecific hypersensitivity reaction. It will be interesting to see if rare skin conditions such as EAC reported in association with COVID‐19 vaccination will reappear during the ongoing worldwide booster campaign.

## AUTHOR CONTRIBUTIONS

All authors take public responsibility for the content of the work submitted for review, and gave their final approval for submission. Alexander Kreuter, Jimmy Jos Puthussery, Julia Hyun and Valentina Laura Müller contributed to the conception of the study. Jimmy Jos Puthussery and Julia Hyun contributed to the acquisition, analysis of data, and drafting of the manuscript.

## CONFLICT OF INTEREST

The authors declare no conflict of interest.

## Data Availability

The data that support the findings of this study are available from the corresponding author upon reasonable request.
